# Tuberculosis Peritonitis Resulting in Small Bowel Obstruction

**DOI:** 10.7759/cureus.68665

**Published:** 2024-09-04

**Authors:** Hannah Clayton, Mark Miller

**Affiliations:** 1 General Surgery, DeBusk College of Osteopathic Medicine, Lincoln Memorial University, Memphis, USA; 2 General Surgery, Methodist Le Bonheur Germantown Hospital, Memphis, USA

**Keywords:** non-caseating granulomas, mycobacterium tuberculous, gastrointestinal stricture, omentectomy, right-sided hemicolectomy, peritoneal tuberculosis (tb), bowel obstruction, tuberculosis peritonitis

## Abstract

Tuberculosis (TB) peritonitis resulting in a small bowel obstruction is uncommon and can be a challenging infectious disease to diagnose. It often has an insidious onset with non-specific symptoms. Today we report a rare case of a 30-year-old woman who recently traveled to Vietnam and presented with worsening upper and lower gastrointestinal symptoms. CT scan revealed an ill-defined mass in the terminal ileum with prominent mucosal enhancement and wall thickening, which ultimately led to subsequent colonoscopy and Quantiferon Gold testing, revealing a positive result. Biopsy of the mass demonstrated noncaseating granulomatous colitis with rare acid-fast positive bacillus consistent with mycobacterial infection. As a result, the patient was ultimately initiated on antituberculosis therapy. Shortly thereafter, she was readmitted with clinical features suggestive of a bowel obstruction. The patient was managed with supportive care and did not require surgical intervention. However, approximately two months following the readmission, she presented to the emergency department once again with a mechanical bowel obstruction that ultimately required surgery. This case underscores the importance of TB testing in patients with insidious, worsening gastrointestinal symptoms and highlights the potential complications of TB peritonitis, even in those undergoing antituberculosis treatment.

## Introduction

Tuberculosis (TB) is as a chronic granulomatous disease that can affect any part of the body, though it most commonly targets the lungs, bones, intestines, and lymphatic tissue [1]. Despite a general decline in TB incidence, extrapulmonary TB has been on the rise over the last decade and is associated with a high rate of morbidity and mortality [2]. Abdominal TB, a subset of extrapulmonary TB, encompasses four forms: nodal, visceral, gastrointestinal (luminal), and TB peritonitis, otherwise known as peritoneal tuberculosis (PTB) [3]. Of these, PTB accounts for approximately 0.9% of all TB cases and is characterized by inflammation of the parietal or visceral peritoneum caused by *Mycobacterium tuberculosis* [4,5]. Diagnosing PTB is particularly challenging due to its nonspecific clinical manifestations, which may include asthenia, nausea, vomiting, weight loss, abdominal pain, ascites, and other symptoms, combined with the absence of specific laboratory and radiologic features [1,4]. Ascitic fluid analysis is the most beneficial nonoperative identifying method for PTB [4]. However, not every patient will present with ascites, necessitating the use of other diagnostic modalities such as CT scan imaging, histopathological examination, and Quantiferon Gold testing, as demonstrated in this case [4]. Due to the subtly progressive symptoms, it is not uncommon for symptoms to persist for six months or more before patients seek medical attention, which was also observed in this case [1,6]. In addition, the possibility of abdominal TB mimicking other abdominal disease processes may ultimately lead to potential delays in diagnosis [4]. Although management of PTB typically involves antituberculosis drugs, surgical intervention may be necessary with the presence of features such as bowel obstruction [3]. This case report discusses a 30-year-old woman diagnosed with PTB after experiencing vague abdominal symptoms for several months. This case further reinforces the critical importance of a thorough history and workup, as well as the complications that can arise from PTB.

## Case presentation

A 30-year-old Vietnamese woman, previously in good health, presented to the hospital with symptoms including nausea, vomiting, and progressively worsening generalized abdominal pain radiating to her back. This pain, which had been intermittent since her return from Vietnam three months ago, was accompanied by early satiety, abdominal fullness, fatigue, weight loss, and reduced oral intake. She reported no chest pain, fever, or diarrhea. Her medical history was unremarkable except for an appendectomy at age 9.

The patient was afebrile, with vital signs within normal limits, and laboratory tests showed a mildly elevated leukocyte count of 10.99 with a lipase of 38, possibly ruling out pancreatitis. She was treated with analgesics and antiemetics while further diagnostic workup was underway.

**Figure 1:**
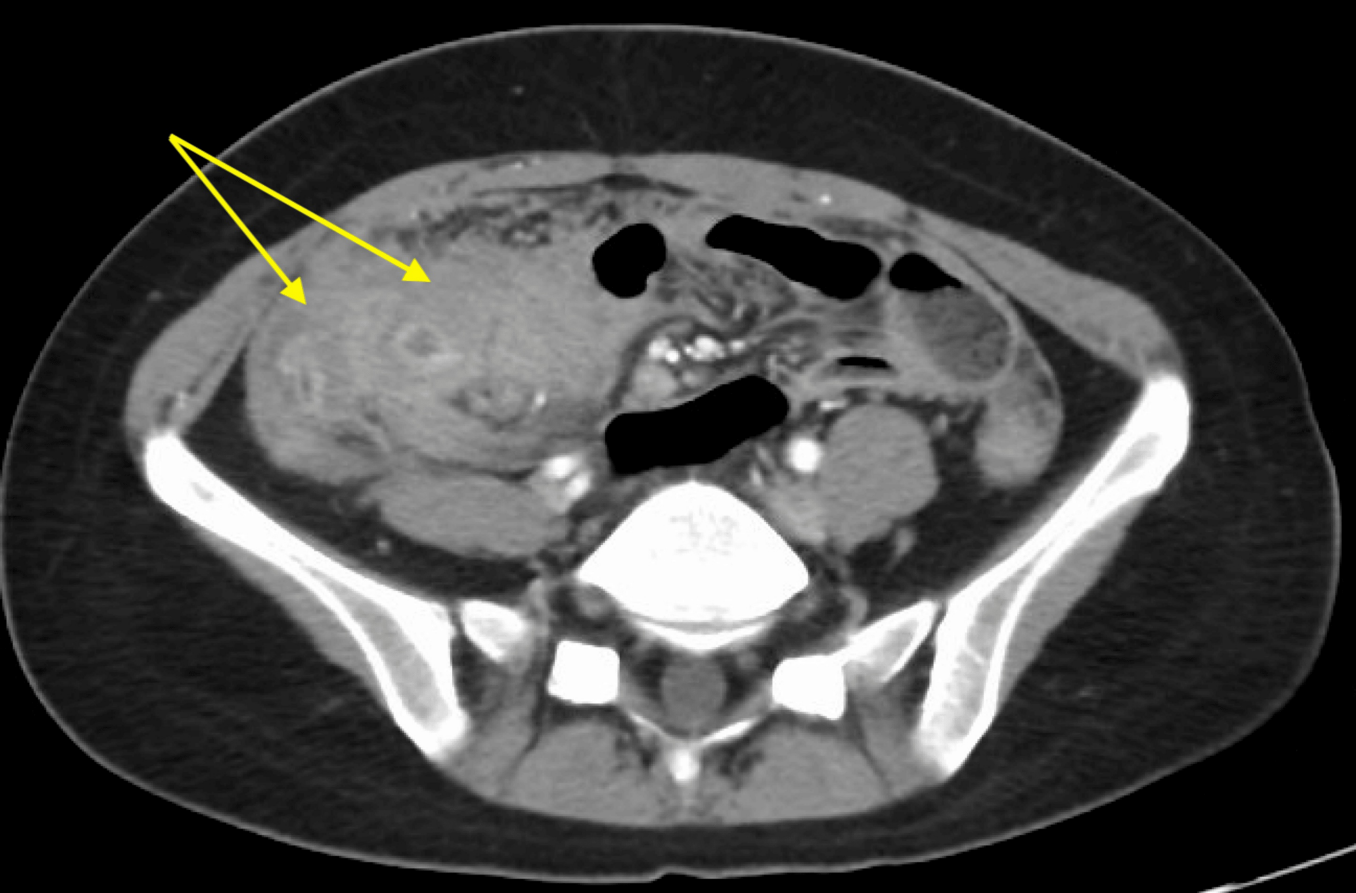
Axial view of CT abdomen and pelvis demonstrating inflammatory change

**Figure 2:**
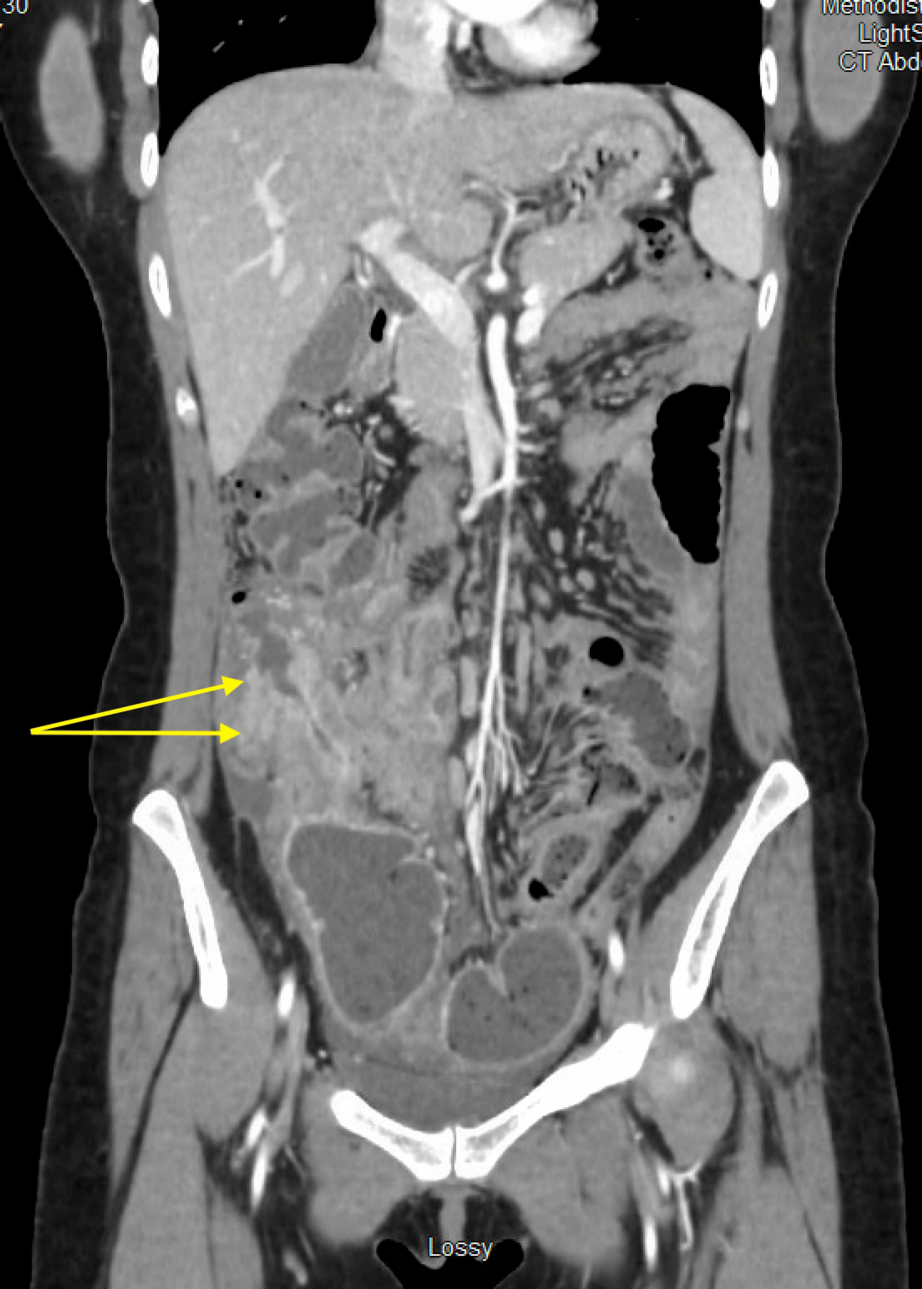
Coronal view of CT abdomen and pelvis demonstrating inflammatory change

On examination, her abdomen was non-distended but markedly tender to palpation throughout, with active bowel sounds in all quadrants and evidence of guarding, but without rebound tenderness. A CT scan of the abdomen revealed an ill-defined mass in the terminal ileum with prominent mucosal enhancement and wall thickening. Additionally, the scan showed diffuse nodularity of the omentum, along with ileocolic and mesenteric lymphadenopathy, raising concern for possible peritoneal carcinomatosis (Figures 1, 2). It remained uncertain whether the inflammatory mass was due to inflammatory bowel disease or a neoplastic process.

**Figure 3:**
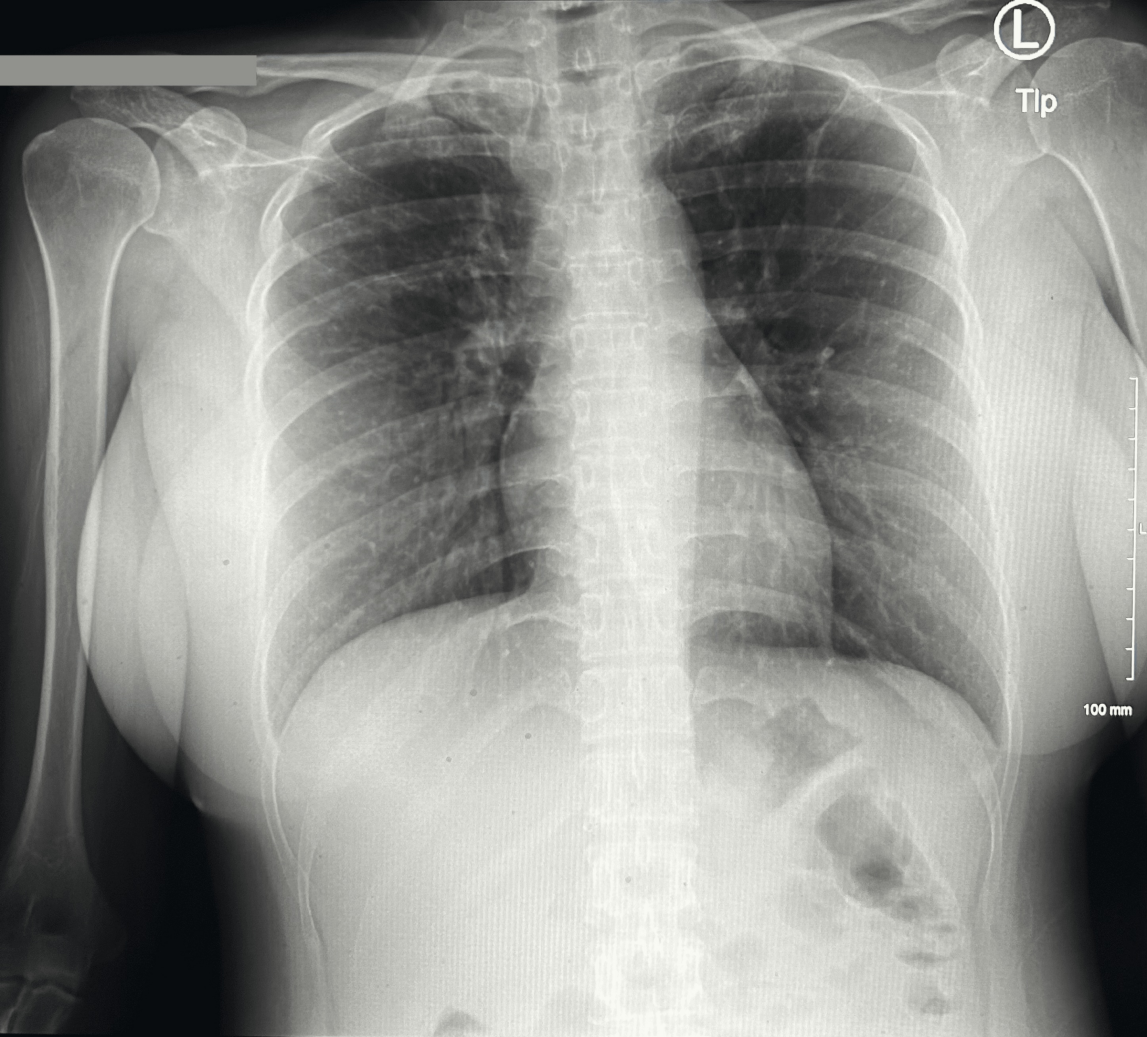
Chest X-ray demonstrating no evidence of active TB TB, tuberculosis

**Figure 4:**
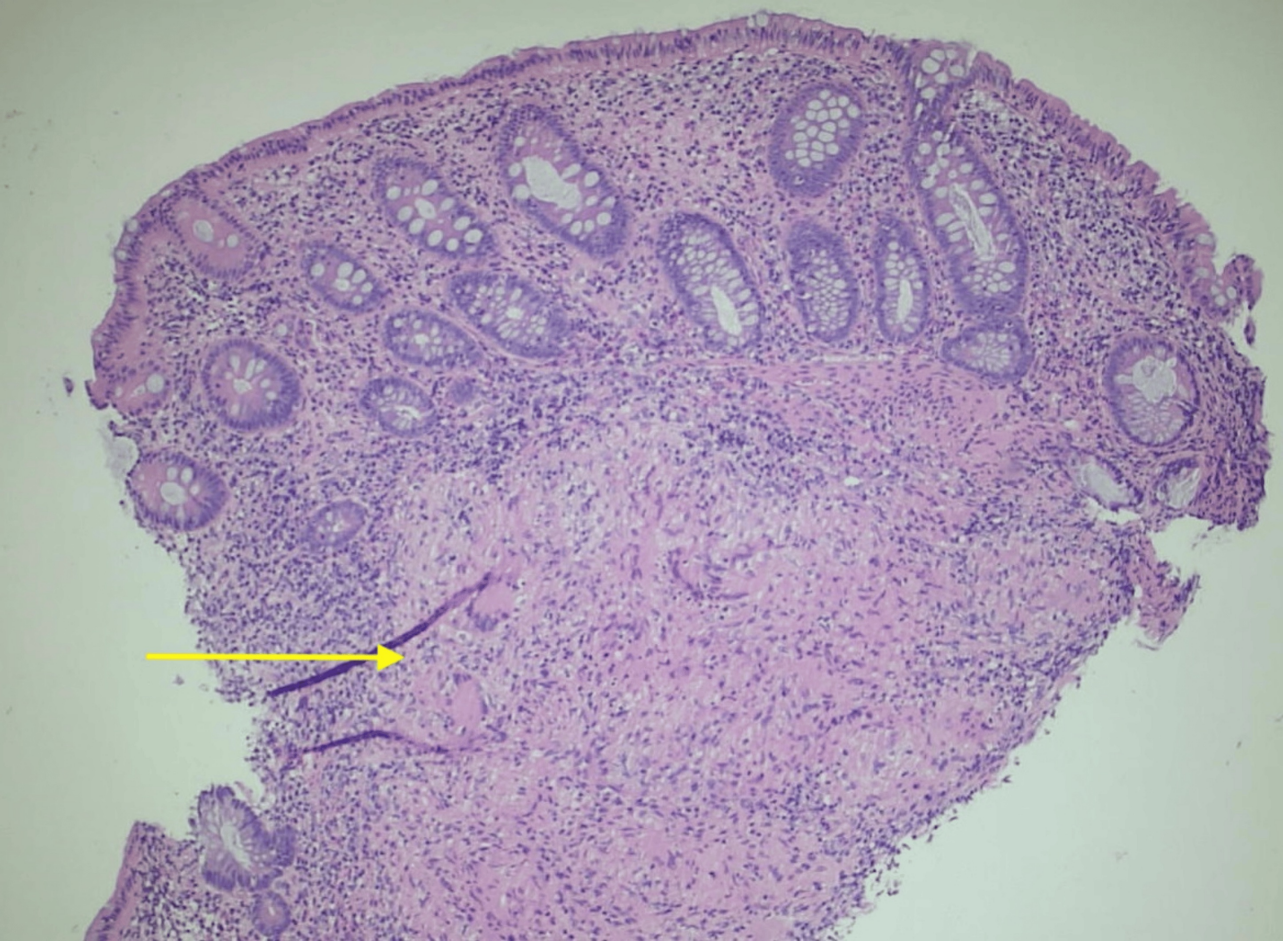
Ascending colonic biopsy demonstrating granulomatous inflammation in the lamina propria

**Figure 5:**
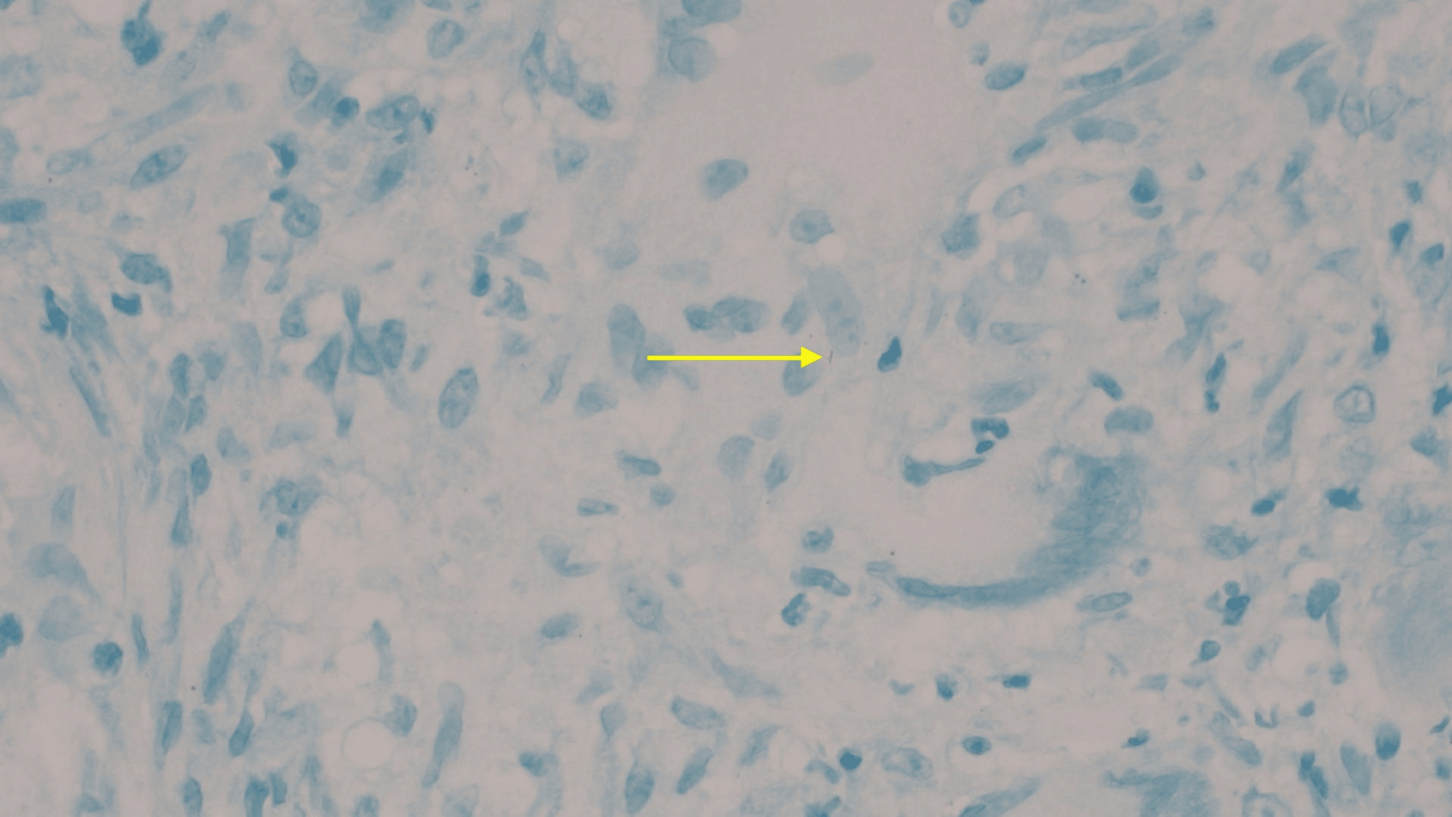
Acid-fast bacilli stain demonstrating rare acid-fast bacillus

Based on the CT scan results, gastroenterology (GI) performed a colonoscopy, revealing a benign inflammatory polypoid mass in the ascending colon that was lobulated and friable, which was subsequently biopsied. Additionally, interventional radiology conducted a CT-guided core needle biopsy of the omentum, and stool cultures were obtained, both of which later tested positive for TB. A Quantiferon Gold test was also positive, while the carcinoembryonic antigen tumor marker was normal at 1.4. Despite these findings, a chest X-ray showed no evidence of active TB in the lungs (Figure 3). Infectious disease specialists noted elevated inflammatory markers with an erythrocyte sedimentation rate of 96 and a C-reactive protein of 30. The pathology report from the ascending colon biopsy identified noncaseating granulomatous colitis with a rare presence of acid-fast positive bacilli, consistent with a mycobacterial infection (Figures 4, 5). The diagnosis was PTB, and a treatment regimen managed by the County Health Department and Infectious Disease consisting of rifampin, isoniazid, pyrazinamide, and ethambutol was initiated before the patient’s discharge.

Approximately one week later, the patient was readmitted with complaints of abdominal pain similar to previously, increased nausea and vomiting, constipation without the ability to pass flatus, fever, fatigue, and generalized weakness. Physical examination revealed abdominal distention and tenderness. A repeat CT scan of the abdomen confirmed the previous findings and additionally showed a partial small bowel obstruction. The patient received supportive care, including intestinal decompression with nasogastric tube insertion, NPO (nil per os) status, fluid resuscitation, pain management, and a brief course of steroids. Due to her NPO status, infectious disease temporarily paused her oral antituberculosis regimen. The partial obstruction resolved within approximately four days and surgical intervention was deemed unnecessary. The patient was able to pass flatus and had multiple bowel movements. She was monitored for over 48 hours on an oral diet with oral medications after the obstruction resolved, during which she gradually resumed a normal diet. The patient resumed her original oral TB medication regimen and was subsequently discharged home.

**Figure 6:**
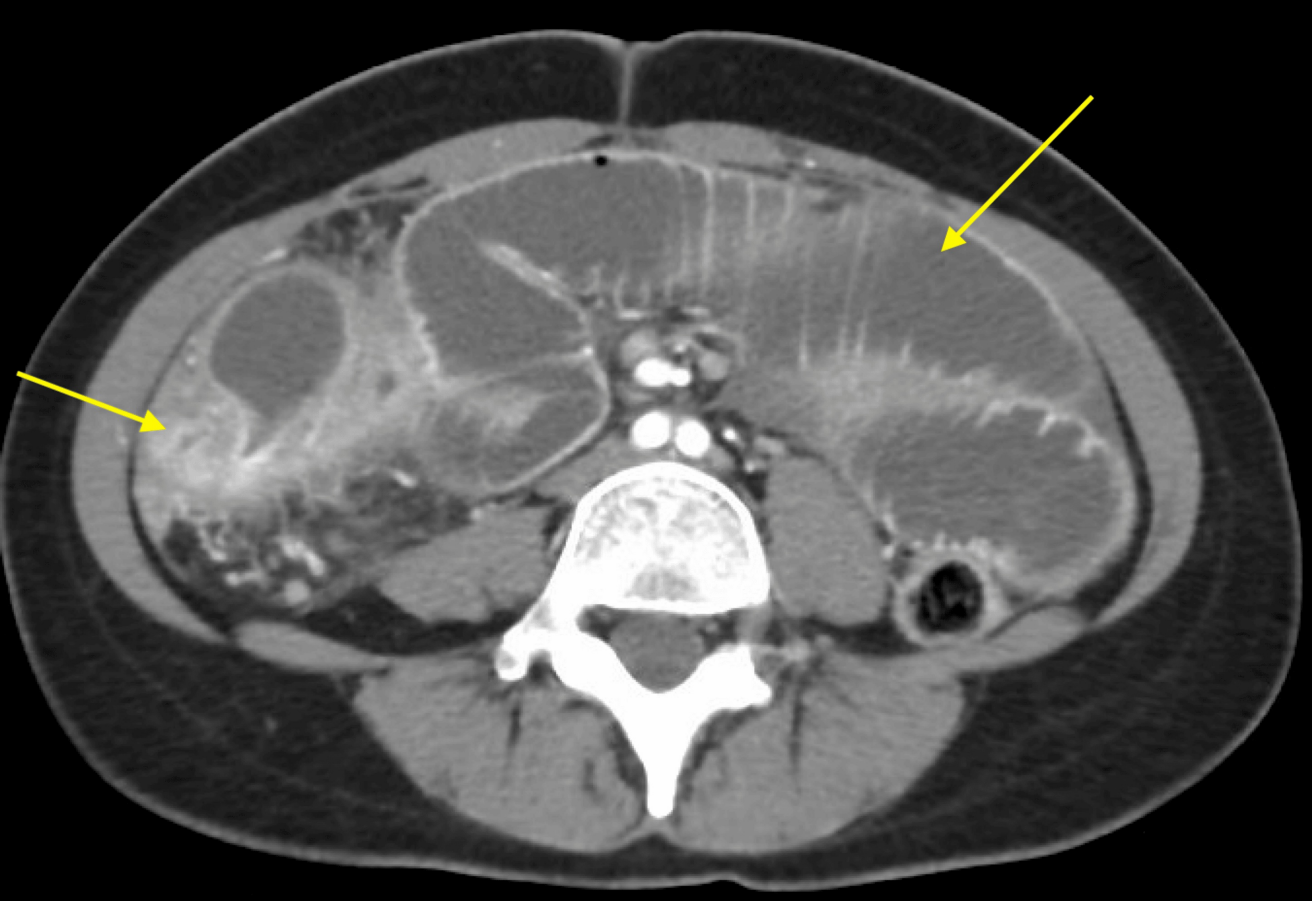
Axial view of CT abdomen and pelvis demonstrating inflammatory change and dilation of colon

**Figure 7:**
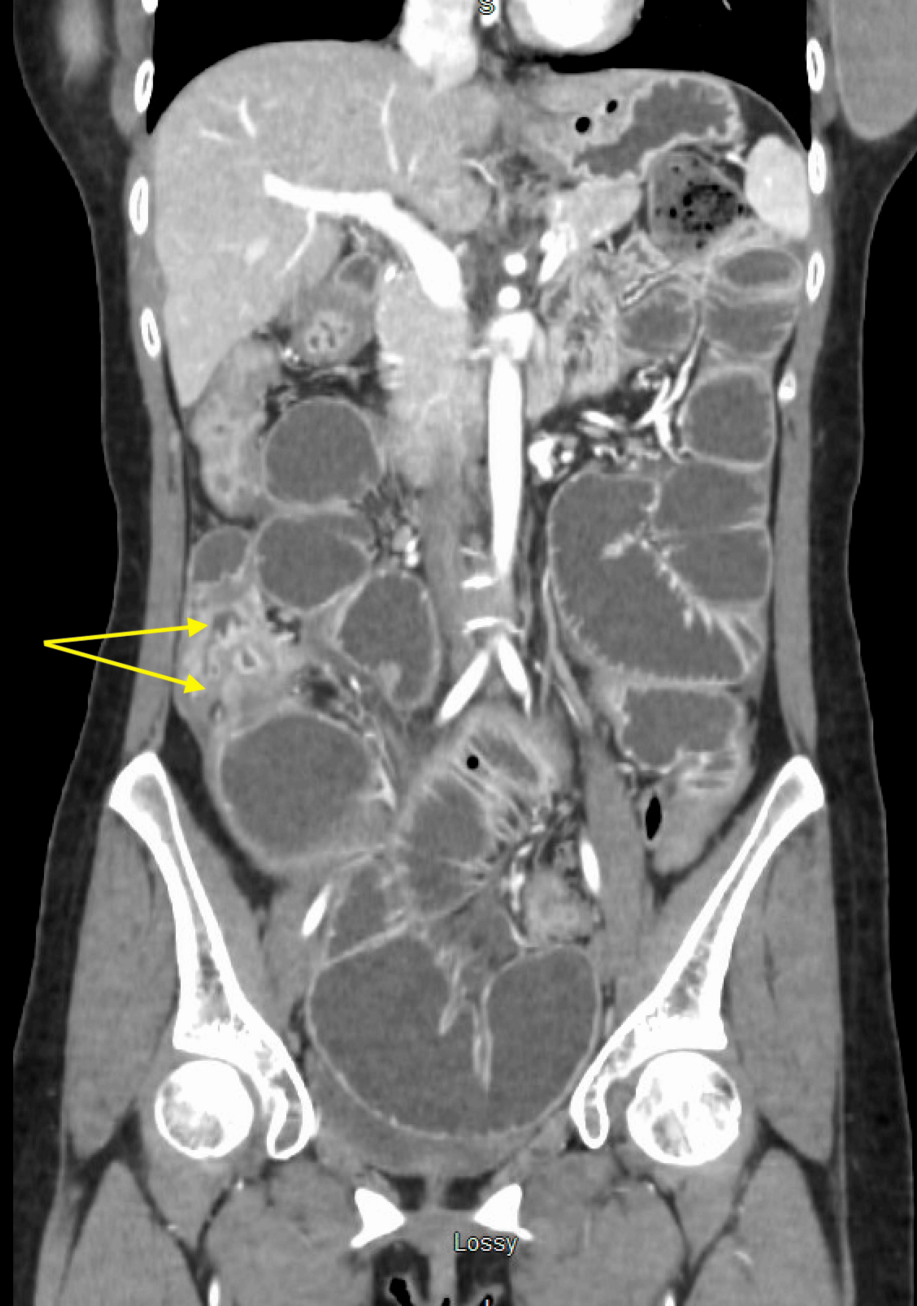
Coronal view of CT abdomen and pelvis demonstrating inflammatory change

She was readmitted a few months later with clinical signs and symptoms of a bowel obstruction. The patient received the same supportive care as before but without a course of steroids. CT of the abdomen and pelvis demonstrated a high-grade mechanical small bowel obstruction at the terminal ileum along with a stricture of the descending colon and re-demonstration of mesenteric lymphadenopathy, omental modularity, and peritoneal implants. It additionally demonstrated submucosal edema, mucosal hyperemia, and regional inflammation along with dilation of colons (Figures 6, 7). Once again, general surgery and infectious disease continued to follow the patient throughout her hospital admission. She was taken to the operating room the following day by general surgery for exploratory laparotomy. Upon exploration, there was diffuse peritoneal disease consistent with PTB. The bowels were run in its entirety. There were mainly small nodules within thin adhesion formation, and the omentum was densely adherent to the abdominal wall in the pelvis. In addition, there were nodules throughout the small bowel but no intra-luminal masses were palpated. There was a loop of bowel adherent to the ileocecal valve toward the terminal ileum along with a large, palpable mass that was both intraluminal and into the mesentery. These adhesions were meticulously taken down. Furthermore, there was a smaller mass in the ascending colon causing a second stricture. Due to the extent of the localized nature of the obstructive disease to the right colon, the decision was made to perform a right hemicolectomy with primary anastomosis due to the remaining bowel appearing health. An omentectomy was also performed due to the severe disease in the omentum. Within a week, after tolerating a normal diet and having bowel movements, she resumed her TB medication and was discharged home. Following discharge, she continued to be observed and monitored by the County Health Department. Additionally, she received routine outpatient follow-up with general surgery, beginning with an initial postoperative visit two weeks later.

## Discussion

PTB is notably rare in the United States, accounting for only about 6% of all TB cases [3]. PTB presents a diagnostic challenge due to its insidious onset and wide range of nonspecific symptoms [1]. Common symptoms include abdominal pain, distention, weight loss, and fever, which can progress subtly and mimic other abdominal pathologies such as neoplastic diseases, congestive heart failure, or liver cirrhosis [4,6]. Consequently, PTB is often diagnosed only after complications, such as bowel obstruction, arise [5]. Reports indicate that small bowel obstruction due to TB ranges from 12-60% and is the leading complication of PTB [5]. This complication arises from intestinal narrowing, strictures, or adhesions secondary to inflammation [1]. Notably, the terminal ileum and cecum are the most frequently affected gastrointestinal sites, likely due to the presence of abundant lymphoid tissue, reduced digestive activity, physiological stasis, and increased fluid and electrolyte absorption [1]. 

The diagnosis of PTB is further complicated by the variety of diagnostic tests available and the expertise required to interpret test results accurately [3]. There are many factors that may contribute to the delay in a patient seeking medical care, as seen in this patient, where a case of PTB resulted in a small bowel obstruction with no signs of pulmonary disease. Importantly, delayed initiation of therapy in PTB is associated with higher mortality rates, with untreated PTB having a mortality rate as high as 50-60% [2].

TB can spread to the peritoneum through hematogenous or lymphatic routes, contaminated food, particularly unpasteurized dairy in the case of *Mycobacterium bovis, *direct extension from adjacent infections, or ingestion of pulmonary sputum [6]. Risk factors for developing abdominal TB include underlying medical conditions such as cirrhosis, diabetes mellitus, renal insufficiency, or malignancy [3]. Alcoholic liver disease and human immunodeficiency virus (HIV) infection are significant risk factors for the development of PTB [6].

Definitive diagnosis of abdominal TB consists of histological visualization of caseating granulomas or by identification of *Mycobacterium tuberculosis* on tissue [1]. According to the World Health Organization (WHO) guidelines, it is recommended to treat abdominal TB with two months of rifampin, isoniazid, pyrazinamide, and ethambutol, followed by four months of rifampin and isoniazid, with some cases requiring surgical intervention, as observed in this case [3]. Intestinal obstruction secondary to TB is considered a complicated case and is typically managed surgically, with the specific procedure determined by intraoperative findings [1]. Depending on whether or not additional complications are involved, possible surgical interventions include stricturoplasty, segmental small bowel resection, perforation repair with or without a protective stoma, ileotransversostomy, right hemicolectomy, or the release of adhesions [1]. This patient ultimately required surgical intervention with a right hemicolectomy and omentectomy due to the extent and severity of the disease.

This case highlights the unique presentation of PTB and emphasizes the importance of differential diagnoses in a patient with unexplained abdominal symptoms, particularly those that persist over an extended period of time. Additionally, it illustrates potential complications that may arise secondary to PTB.

## Conclusions

While PTB is rare, its incidence and prevalence have been increasing. It often necessitates a high degree of clinical suspicion, especially in patients with a gradual onset of symptoms and non-specific manifestations, particularly if they are at increased risk, have recently traveled to TB-endemic areas, or present with bowel obstruction. Due to its potential to resemble other conditions, a detailed patient history and comprehensive diagnostic evaluation are crucial. Treatment varies on the patient's presentation and should be tailored to the individual patient. For instance, in this case, the patient required antituberculosis medications managed by the County Health Department as well as surgical intervention to address complications. Nonetheless, each treatment plan should be customized based on the patient’s specific presentation.
